# A Few Notes on Some of the Principal Centers of Veterinary Education in England and on the Continent

**Published:** 1887-04

**Authors:** A. W. Clement


### Art. XIV.—A FEW NOTES ON SOME OF THE PRINCIPAL CENTERS OF VETERINARY EDUCATION IN ENGLAND AND ON THE CONTINENT.

BY A. W. CLEMENT, V. S.

It is now considered almost a necessary part of a medical student's education to spend a certain time abroad in the furtherance of his studies, under men of world-wide reputation, who have an almost unlimited amount of material at their disposal for teaching purposes.

If in the present limited state of competition, in the veterinary profession, it is not absolutely necessary, it is none the less desirable that the veterinary student should make his education as complete as possible, the more because at present the facilities offered for practical study by the colleges in America are not to be compared with those of the old world.

We shall confine our remarks to a description of the three largest, and therefore, representative colleges of the countries to which they belong, viz.: The London school for England, Ireland and Scotland; the Berlin school for Germany, and the Alfort school for France. Perhaps to the majority of American students the London school alone will have any practical interest, both from the language spoken and on account of the advantages to be gained from the possession of the Royal College diploma. To others, however, who may wish to do some special work, or who wish to compare the work of the leading men in the different countries, a short account of these schools may be of interest.

The winter session at Berlin begins about the first of October and finishes the middle of March; the summer session begins about the first of May and ends the last of July. If one leaves New York about the middle of March, the time when the winter session closes at most of our American schools, he will arrive in Berlin in good time to take advantage of the private courses given at Professor Virchow's laboratory during the spring recess. This laboratory, the pathological institute connected with the Charity hospital, is immediately opposite the veterinary school and affords splendid opportunities for working at pathological anatomy. At the pathological institute connected with the veterinary school will also be found abundant material, though no instruction is given here during the recess. The clinics go on, however, the same as during the session, though the professors may not always be present. Then at the central "viehof," or slaughter house, of course there is as much material at one time as at another, so that one will find enough to do to keep him very busy even during vacation. The pathological teaching at the Charity goes on from seven till ten, seven mornings in the week and includes much instruction in comparative as well as human pathology.

The clinics at the veterinary school begin at ten o'clock and finish at twelve. Two afternoons each week should be devoted to the viehof, where a vast amount of informatian may be gained. All of the animals used for human food in Berlin are slaughtered at this establishment, and every carcass is skillfully inspected before it is allowed to leave the premises; about 150 persons being engaged in the service, 25 of whom are veterinary surgeons. The number of animals consigned daily to the vats shows either that the animals are much more diseased than are ours at home, which is not true, or that we consume a great deal of meat totally unfit for human food, which is true. The pathologist to the institution, Mr. Dunker, is very obliging, and will give one all the assistance necessary. Permission to work here must first be obtained from Dr. Hertwig, chief of the inspection service, but there is no trouble in getting it, as he is a most obliging gentleman. Much original work is done in Mr. Dunker's laboratory, he having first detected the presence of actinomyces in pork. The other afternoons may well be spent at the veterinary school, in the assistants' laboratories, at the pathological institute, or in the clinics, which are held from four till five.

One can hardly form any idea of the immense amount of material at command in almost every branch.

There are four clinics going on at the same time, each in charge of a specialist. There is the medical clinic, the surgical clinic, the smaller animals clinic and the polyclinic. In the surgical wards one may see on an average from 125 to 150 horses as resident patients, and in the medical clinic about the same number; in the polyclinic from 50 to 60 larger animals daily. The smaller animals clinic is divided into two parts, the inner and the outer. In the inner clinic are on an average 75 resident patients, mostly dogs.

I forgot to mention the ambulance clinic, where a certain number of students are taken daily, under charge of a special professor, to visit sick animals at private residences, mostly in the suburbs.

English and American veterinary surgeons complain of college competition, but I don't know what they would say if they were in Berlin. It seems as if everybody must send their sick animals to the college, and I have seen dogs there for a long time, belonging to some of the highest noblemen.

Everything is specialized, and consequently the teaching is the best possible. It is thoroughly practical also, impressions to the contrary notwithstanding. Nowhere, either in veterinary or medical hospitals, is the teaching more systematically carried out from the time the patient enters the hospital until he goes away from there alive, or from the pathological institute in pieces. The daily records of the cases are insisted upon, the diagnosis, treatment and prognosis entered in a special book, and if the animal dies, the post-mortem diagnosis must be made and recorded by the pathologist, who is not supposed to know anything about the clinical diagnosis. The clinical professor takes his class to the post-mortem room and views the organs, pointing out as nearly as possible the relation between the lesions found and the ante-mortem symptoms. On three mornings in the week the pathologist demonstrates the morbid anatomy specimens, and once or twice a week gives instruction in the method of making post-mortems, but in addition to this the students in groups make post-mortems under the direction of the assistants.

About three hundred autopsies are made here yearly upon horses, and I would not like to say how many upon the smaller animals. Much material in the smaller animals goes to waste because the assistants have not time to attend to it.

In the surgical clinic the operations are generally performed by the professor or his assistant, thereby differing from the Alfort school, where the students do nearly everything. Two or three operations a day are the rule. They are very thoroughly performed and the results are sometimes wonderful. Dry dressings are exclusively used, and during the operation the wound is frequently irrigated with corrosive sublimate water. The dry dressings are iodoform and corrosive sublimate with cellulose. A great variety of operations may be seen here. Perhaps they do not cut as much as the French do, but the preliminary work and the dressing is, I think, much better done. As a preliminary to an operation about the foot, as, for example, removal of diseased cartilage and bone, the horse is made to keep his foot in a bath of corrosive sublimate water for at least twelve hours previous to the operation. In France, on the other hand, the horse is thrown down immediately it is decided to operate, and the cutting begins without any further ceremony. The medical clinic in Berlin is very well conducted and so is the smaller animals clinic.

The buildings at the Berlin school are scattered over about thirty acres of land in the center of the city, and, as I said before, opposite the Charity, the largest hospital in the city. With its surroundings it forms a little village within a large city, and it is very pleasant to walk about the well-kept grounds upon a summer's day, quite apart from the busy world without. In one part of the grounds is the veterinary physiological institute, presided over by Professor Monk, who has acquired such a reputation in the localization of brain centers by his experiments on dogs, which he still keeps up. Opposite, the anatomical institute of the university, at the head of which is Waldeyer, probably the leading histologist of the day. Then the hospitals between this and the pathological institute at the other end of the grounds, and Professor Virchow's laboratory, just across the street at the Charity. One must work to see much here, yet the surroundings make a man work, and he will always find plenty ready to help him. If possible, let anybody who intends spending any time in Berlin have some special object in view, for he will find abundant material in almost any class of medicine or surgery.

At the London school the system of teaching is altogether different, but the professors are very enthusiastic and seem never tired of doing all in their power to help the student. They have not the means, however, and therefore can not carry out specialized teaching to such an extent as is done in Berlin; nor do I know that they would if they could, for they seem to have the impression, and not without reason, that a man's influence is better as a teacher when he is a fairly good all-around man, than when he knows something exceedingly well, perhaps at the expense of his general professional information. One thing is certain however, that nowhere will you see as many horses examined for soundness as in London. You can see as many in London in one day sometimes as you can in Berlin in two weeks, or perhaps in a month. The medical clinic in London is also good but there is very little surgery. The professors are not specialists. Each of the three clinical teachers has his day for receiving, and all the cases which come to the infirmary on that day are his cases, be they surgical or medical, large or small animals. If an animal dies and a post-mortem is made, it is done under the direction of the professor who had charge of the case. The material for post-mortems at the college is very limited and for want of time much of it goes to waste; probably this will be remedied before long, as at present they have the will if not the way. If the knackers' establishment, not far away, could be brought into use there would be abundant material for pathological investigation.

The Alfort school, situated about four miles from Paris, offers especial facilities for seeing foot surgery; hardly a day passes but what eight or ten horses are operated upon, and nearly all upon the foot and hock. Most of the horses go away as soon as operated upon, there being accommodations for about 60 only in the infirmary. The clinical teachers do not divide the work here, but as in London each on his day receives all that comes. If a horse is to be operated upon, the operation is performed by the students under the direction of the assistants, except in special cases, when the professor may do the most difficult part. There is no preliminary bath, and tow saturated with tr. iodine is used as dressing, in place of corrosive sublimate. They do not hesitate to go to the bottom of a wound and remove all diseased portions, even if healthy tissue has to be sacrificed. I several times saw the whole sole pulled off, and twice I saw the navicular bone exposed.

The dog hospital is perhaps as large as at Berlin, but not nearly as much attention is paid to it. Large new laboratory buildings have just been erected, but there does not seem to be a great abundance of material. The shoeing forge is very fine, and in this it is ahead of Berlin. Living expenses are about the same in London and Berlin, but in the latter place one needs a first-class stomach to digest the food. In Paris it is much more expensive living.

If I were to do the trip over again and had twelve months at my disposal, I should spend eight in Berlin, three in London and one at Alfort.

